# Ecotoxicity testing of airborne particulate matter—comparison of sample preparation techniques for the *Vibrio fischeri* assay

**DOI:** 10.1007/s10653-021-00927-w

**Published:** 2021-04-16

**Authors:** Nora Kováts, Katalin Hubai, Tsend-Ayush Sainnokhoi, András Hoffer, Gábor Teke

**Affiliations:** 1 Institute of Environmental Sciences, University of Pannonia, Egyetem str. 10, Veszprém, 8200 Hungary; 2 School of Veterinary Medicine, Mongolian University of Life Sciences, Khan-Uul District, Zaisan, Ulaanbaatar, 17042 Mongolia; 3 MTA-PE Air Chemistry Research Group, Egyetem str. 10, Veszprém, 8200 Hungary; 4 ELGOSCAR-2000 Environmental Technology and Water Management Ltd., Balatonfuzfo, 8184 Hungary

**Keywords:** Airborne particulate matter, Diesel exhaust, *Vibrio fischeri*, Direct contact test

## Abstract

The bioassay based on the bioluminescence inhibition of the marine bacterium *Vibrio fischeri* has been the most widely used test for the assessment of airborne particulate matter ecotoxicity. Most studies available use an extract of the solid sample, either made with water or organic solvents. As an alternative, a whole-aerosol test is also available where test bacteria are in actual contact with contaminated particles. In our study, different extraction procedures were compared to this direct contact test based on the *V. fischeri* assay and analytical measurements. The lowest PAH content and the highest EC_50_ were determined in water extract, while the highest PAH amount and lowest EC_50_ were measured in dichloromethane, hexane, and dimethyl-sulphoxide extracts. EC_50_ of the direct contact test was comparable to that of the methanol extract. Our results suggest that the sensitivity of the direct contact test equals to that of extraction procedures using organic solvents, moreover, it is mimicking an environmentally realistic exposure route.

## Introduction

Airborne particulate matter (PM) is grouped as coarse, fine, and ultrafine particles (UFPs) with aerodynamic diameters of 2.5–10 μm (PM10), < 2.5 μm (PM2.5), and < 0.1 μm (PM0.1), respectively. These particles consist of a complex cocktail of potentially toxic compounds (Gualtieri et al., 2009). Potentially, toxic chemicals associated with PM emissions include for example heavy metals (Guo et al., 2021) or polyaromatic hydrocarbons (Liu et al., 2014; Najmeddin & Keshavarzi, 2019). While PM10 and PM2.5 concentrations are often monitored (Huang et al., 2020; Yu et al., 2020), the risk of UFP fraction has been recognised quite recently. Smaller particles bind relatively higher amount of hazardous chemicals as they have specific surface area (Mbengue et al., 2015) and therefore pose higher ecological risk (Landkocz et al., 2017). These particles penetrate deep into the respiratory system and elucidate adverse health effects even at low concentrations (Ali et al., 2019). In addition to chemicals, potentially hazardous and/or pathogenic biological agents might also be carried by particulates such as viruses (Zoran et al., 2020) or bacteria (Xu et al., 2019).

Chemical analysis is often coupled with ecotoxicological tests, which quantify the overall toxic effect on selected test organisms and provide information about the bioavailability of the toxicants (Kessler et al., 2012). As such, actual ecological risk of the sample in question can be estimated (Klimkowicz-Pawlas et al., 2019).

While human health effects or airborne contaminants have been widely discussed (e.g. Dastoorpoor et al., 2019; Kelly & Fussell, 2015; Khaefi et al., 2017; Naghizadeh et al., 2019), much less information is available regarding the effects on the non-human biota.

When ecosystem-level impacts are to be addressed, a battery of bioassays should be employed (de Sá Salomao et al., 2020), involving test species which represent important structural and/or functional elements (guilds) of the recipient ecosystem and show different response (Alvarenga et al., 2008; Luo et al., 2020).

Wang et al. (1998) used a test battery involving the *Photobacterium phosphoreum* luminescent bacterium assay, *Dunaliella tertiolecta* (a dinoflagellate green alga), *Brassica chinensis* (Chinese white cabbage), and *Lolium perenne* (rye grass) to take a comparative study on the water extract of urban dust samples from Hong Kong and London. While no significant correlation was found between the tests, the luminescent bacteria assay showed good correlation with Pb and Zn, and *D. tertiolecta* with the exchangeable Pb content. In the study of Barbosa et al. (2013), the potential hazard of size fractionated biomass ashes was evaluated. Test species involved marine (the bacterium *Vibrio fischeri*, the micro-crustacean *Artemia franciscana* and the microalgae *Phaeodactylum tricornutum*) and freshwater (the micro-crustacean *Daphnia magna* and the microalgae *Selenastrum capricornutum*) organisms. In this study, *V. fischeri* proved the most sensitive species.

Single-species tests have been used to study the chronic effects and mode of action of PM2.5 on the nematode *Caenorhabditis elegans* model species (Chung et al., 2019, 2020).

The bioassay based on the bioluminescence inhibition of the marine bacterium *V. fischeri* has proven an ideal tool for screening potential ecotoxicity of atmospheric PM (Roig et al., 2013), due to numerous reasons. This test has reported sensitivity to toxic compounds typically found in PM samples such as PAHs and heavy metals (Palma et al., 2014). Toxic compounds will cause the inhibition of the NAD(P)H: FMN oxidoreductase and luciferase enzyme system, which is reflected in the rapid decrease of light emittance of the bacterium. This decrease is proportional to the strength of the toxicant, giving an easy-to-quantify end point. The assay is recommended for the initial screening of samples with unknown toxic potential (Menz et al., 2013). Test results are also predictive for acute toxicity in other organisms (Weltens et al., 2014).

Several commercial systems are in use which are based on this phenomenon (reviewed by Kokkali & van Delft, 2014). The test organisms (*V. fischeri* strains) are available in liquid-dried or freeze-dried kits, avoiding the laborious task of maintaining a stock culture. Also, the test is automatised, as such, human errors can be minimised. The test is relatively easy-to-perform and requires a short exposure (with a maximum exposure of 30 min, a sample can be evaluated). Considering all these benefits, the test has been widely recommended to measure the toxicity of water, soil, or sediment samples (American Society for Testing & Materials, 1995).

Additional benefit of the assay is that low sample quantity is needed which is extremely important if airborne particulate matter is to be analysed. Airborne emissions are generally collected on a filter, which limits the quantity of the sample.

The test has been successfully employed in diverse environmental matrices (reviewed by Abbas et al. 2018; Girotti et al. 2008; Ma et al. 2014). *V. fischeri* was already used in the 1960’s to evaluate air quality (Serat et al., 1965, 1967). Since then, a wide range of bioluminescence-based tests and biosensors have been designed for air pollution assessment (reviewed by Kováts & Horváth, 2016).

The original protocol (Bulich & Isenberg, 1981), which is followed by the ISO 11348 water quality—Determination of the inhibitory effect of water samples on the light emission of *Vibrio fischeri* (Luminescent bacteria test) international standard, uses an aqueous sample. As such, for solid samples, an extract has to be prepared.

Silva et al. (2015) detected significant toxicity in aqueous extract of ash samples produced by forest fires. Wang et al. (2016) also used aqueous extract to assess seasonal differences in urban atmospheric fine particulate matter (PM2.5) collected in Beijing (China). Roig et al. (2013) performed aqueous extraction of air filters by means of a mild acid microwave digestion in a study covering various areas of Catalonia (Spain). Good correlation was found between toxicity and chemical parameters. Aammi et al. (2017) applied extraction with dimethyl-sulphoxide (DMSO) and ultra-pure water in parallel for characterisation of urban (Istanbul, Turkey) PM2.5–10 samples. While the toxicity levels were below the limit of detection for the majority of the samples, DMSO extracts indicated the seasonal difference in the ecotoxicity of urban samples. In the study of Romano et al. (2020) conducted on PM10 samples collected at a coastal site of the Central Mediterranean, *V. fischeri* tests were carried out from the aqueous extract of the samples. Ecotoxicity results showed good correlation with Dithiothreitol (DTT) results which indicate oxidative potential. One major mode of action of PM-bound toxic chemicals is to cause oxidative stress (Bekki et al., 2016) and *V. fischeri* bacteria have proven sensitive to this stressor (Zhang et al., 2019).

In addition to water extracts, different organic solvents have been in use. Extraction with acetone and hexane was applied in the study of Evagelopoulos et al. (2009) to assess the ecotoxicity of urban (industrial) coarse (2.5–10  µm) and fine (< 2.5 µm) particulate matter. Isidori et al. (2003) applied cyclohexane for extraction to assess spatial pattern of urban air pollution in Caserta (South Italy) while Triolo et al. (2008) applied extraction with acetone and hexane to monitor the impact of PM emitted by the industrial settlement of Milazzo (Italy) on agriculture. In the study of Chang et al. (2013), samples were extracted by n-hexane or dichloromethane/n-hexane mixtures to compare the toxicity of fly ash after incinerating plastic solid waste (PSW) and organic liquid waste (OLW). Verma et al. (2012) compared the ROS (reactive oxygen species)-generating potential of water- and methanol-soluble fraction of urban fine aerosols (PM2.5).

Several studies are available which characterised the ecotoxicity of vehicle emissions using the *V. fischeri* assay. Lin and Chao (2002) used dichloromethane (DCM) and a mixed solvent containing n-hexane and DCM to assess the influence of methanol-containing additive on biological characteristics of diesel exhaust emissions. Similarly, DCM was applied as a solvent in the study of Vouitsis et al. (2009) when PM emitted from light-duty vehicles in different driving cycles was analysed.

Different sample processing procedures, however, make only intrastudy comparison possible as range and concentration of the toxic compounds in the extract will strongly depend on the solvent applied, influencing resulting ecotoxicity (e.g. Corrêa et al., 2017; Verma et al., 2013).

In order to avoid the problem raised by the nature of solvent and extraction procedure applied, direct contact tests might be necessary. In direct contact tests, test organisms are in contact with toxic particles. The Microtox® Solid Phase Basic Test was developed for solid-phase (sediment and soil) testing (Brouwer et al., 1990) and was later proposed as a standard protocol. Stock suspension of the solid sample is prepared, which contains test bacteria. After the exposure period, the solid particles are removed by filtration and toxicity of the water fraction is measured. However, false (lower) light emission reading might occur, as a given portion of the bacteria is supposed to be lost due to their adhesion onto the particles (Ringwood et al., 1997). In order to eliminate or reduce this kind of error, several improvements have been made (Yeo et al., 2015). Burga Pérez et al. (2012), e.g. suggested the Microtox leachate phase assay (MLPA), where amount of bacteria lost is measured using flow cytometry and allows differentiating real ecotoxic and fixation effect.

This assay was used by Ledda et al. (2013) to evaluate professional exposure to basaltic rock dust in the region of the volcano Etna (Italy) and by Goix et al. (2014) to assess the ecotoxicity of fine and ultrafine metallic particles released into the atmosphere.

A specific ‘whole-aerosol test’ was developed by Kováts et al. (2012). The test is based on the kinetic version of the *V. fischeri* bioluminescence bioassay which was especially tailored to carry out toxicity measurements in case of coloured or turbid samples (Lappalainen et al., 1999, 2001). The protocol is available as an ISO standard, too (ISO 21338:2010: Water quality—Kinetic determination of the inhibitory effects of sediment, other solids and coloured samples on the light emission of *Vibrio fischeri* /kinetic luminescent bacteria test/). The kinetic nature of the test implies that light output is continuously recorded for the first 30 s. This kinetic diagram immediately shows if the sample is toxic and can be already used for preliminary assessment (Mortimer et al., 2008). Jarque et al. (2016) used the 30-s kinetic bioassay to evaluate the toxicity of environmental samples. Inhibition is calculated after the preset exposure comparing the initial and final readings, independently from the control.

The ‘whole-aerosol test’ uses a suspension where filter spots are ground in an agate mortar then mixed with high-purity water (MilliQ) by continuous stirring. As such, test bacteria are in fact direct contact with particles. When comparing this assay with the toxicity of aqueous extract gained from the same aerosol samples, it was able to eliminate false toxicity readings caused in case of non-toxic but coloured and turbid samples, such as the extract of summer aerosol (Kováts et al., 2012). It has been used to assess the ecotoxicity of emission of diesel-powered light- and heavy-duty vehicles (Ács et al., 2013; Kováts et al., 2013) as well as urban and rural PM samples (Turóczy et al., 2013).

Our hypothesis implies that extraction procedures, especially those which apply organic solvents, will not represent natural exposure pathways, moreover, organic solvents may extract that fraction of particle-bound contaminants which would not be normally biologically available (DelValls et al., 2004).

The main objective of the study was to compare toxicity results gained by using different extracts and the direct contact version, answering basically two questions: (1) Are there any significant differences in the ecotoxicity of samples prepared by different extraction procedures, also comparing these extracts to the direct contact test? (2) Can the direct contact test be regarded as a good representative of bioassays assessing particulate matter ecotoxicity?

## Material and methods

### Sampling and sample preparation

PM10 samples from the exhausts of a diesel-powered jeep (Euro 4 environmental standard, age 13 years, odometer reading: 256,887 km) were collected with a high-vol sampler (Kálmán System, KS-303) on quartz filter at a flow rate of 32 m^3^h^−1^ for 10 min at idling in a closed premise about 1 m from the tailpipes. The filter was divided into 6 equal parts and processed as described in selected studies with minor modifications:

1. Extraction with methanol (Verma et al., 2012)
2. Dichloromethane (DCM) (Vouitsis et al., 2009)
3. Dimethyl-sulphoxide (DMSO) (Aammi et al., 2017)
4. Hexane (Chang et al., 2013)
5. Water (Wang et al., 1998)
6. Direct contact test (Kováts et al., 2012).
7. Extraction with methanol: One-sixth of sample filter was cut into pieces and then ultrasonically extracted with methanol for 15 min in an ultrasonic bath. The sample was filtered using Polytetrafluoroethylene (PTFE) 0.45 µm pore size filter to remove insoluble materials. Methanol extract was evaporated in nitrogen stream at 40 °C to 1 ml. The extracted sample was diluted before analysis. About 250 µl of extracted sample was added to 14.75 ml DMSO.
8. Extraction with dichloromethane (DCM): One-sixth of filter sample was extracted in 300 ml DCM for 24 h and then evaporated to reduce volume to 1 ml at 40 °C with nitrogen stream. The 1 ml extract was changed with 1 ml DMSO. The diluted extraction was kept in freezer until measurement. 250 µl of original extracted sample was filled up to 15 ml with DMSO.
9. Extraction with dimethyl-sulphoxide (DMSO): One-sixth of filter sample was cut into volume of 15 ml plastic tubes with 2% DMSO and extracted for 15 min in a sonication bath and then centrifuged to separate soluble components from the insoluble ones at 2500 rpm for 10 min. The supernatant was filtered using PTFE 0.45 pore size filter to remove the insoluble parts. The extract was kept in freezer until analysis. Prior to testing, 11.75 ml DMSO was added to 3.75 ml of extracted sample.
10. Extraction with hexane: One-sixth of sample filter was extracted in 300 ml hexane using a Soxhlet apparatus, the supernatant was evaporated at 40 °C with nitrogen stream to 1 ml. Prior to measurement, 250 µl of initial extracted sample was added to volume of 14.75 ml of DMSO.
11. Extraction with de-ionised water: One-sixth of the filter was cut into pieces, placed in a beaker and ultrasonically extracted in 20 ml de-ionised water. Prior to measurement, 1.25 ml of initial extracted sample was diluted with 13.75 ml de-ionised water.
12. Direct contact test: the filter sample was ground in an agate mortar then transferred into a pre-cleaned 4 ml vial with a PTFE-coated spatula. Suspension was prepared adding 2 ml high-purity (MilliQ) water. The suspension was finally diluted to 15 ml.

### Ecotoxicity testing

All samples were processed according to ISO 21338:2010: Water quality—Kinetic determination of the inhibitory effects of sediment, other solids and coloured samples on the light emission of *Vibrio fischeri* (kinetic luminescent bacteria test). Prior to measurement, freeze-dried test bacteria (NRRL-B-11177 strain, supplier Hach Lange Co.) were rehydrated with the reconstitution solution and incubated at 15 °C for 15 min.

Serial dilutions (1:1) for each sample were directly prepared in 2% w/v NaCl (pH7.0 ± 0.2) in 96-well microplate. For the assay, the Ascent Luminometer (Thermo Scientific) was used which is equipped with computer-controlled injectors. After the sample was added to the suspension made of rehydrated bacteria, bioluminescence intensity was continuously recorded for the first 30 s. Luminescence intensity was read again after the preset exposure time (30 min). EC_50_ was calculated from the light inhibition percentages by the Aboatox software provided with the Ascent Luminometer.

### Analytical measurements

Analytical measurements were performed in the testing laboratory at the Laboratory of the ELGOSCAR-2000 Environmental Technology and Water Management Ltd. accredited by the National Accreditation Authority, registration number NAH-1-1278/2015.

The polycyclic aromatic hydrocarbons (PAH) content was determined by gas chromatographic–mass spectrometry (Agilent 6890GC 5973E MSD GC–MS) according to MSZ (Hungarian Standard) 1484-6:2003. For quality control, the glassware was thoroughly cleaned before use, washing with non-ionic detergent and rinsing with ultrapure water. A PAH standard mixture were obtained from Restek Corporation, (U.S., 110 Benner Circle, Bellefonte, PA 16823.). Linearity of the calibration curve was checked in accordance with the accreditation standard.

## Results and discussion

Table 1 gives the comparative values as expressed in the form of EC_50_ (the percentage of the original sample causing 50% of luminescence inhibition).

**Table 1:** Ecotoxicity of different extract vs. the direct contact test

Sample	Methanol	Hexane	DCM	DMSO	Direct contact	Water
EC_50_	12.53	6.79	6.39	9.92	11.97	78.31

The hexane and DCM extracts were the most toxic, EC_50_s fell very close to each other (6.79 and 6.39%, respectively). EC_50_ of the direct contact test was somewhat higher, similarly to that of the methanol extract (11.97 and 12.53%, respectively). Water extract showed considerably lower toxicity, with EC_50_ of 78.31%. In order to evaluate the impact of the bioavailable contaminant fraction of sediments, Baran et al. (2019) also applied the *V. fischeri* test and categorised toxicity as non-toxic (percentage effect ≤ 20%); slightly toxic ≤ 50%; toxic ≥ 50% and highly toxic samples (percentage effect = 100%). According to this classification, extract prepared with organic solvents gave highly toxic results as maximum inhibition in the concentrated sample was 100% while water extract gave non-toxic outcome, with maximum inhibition of 14.5%.

**Fig. 1:**
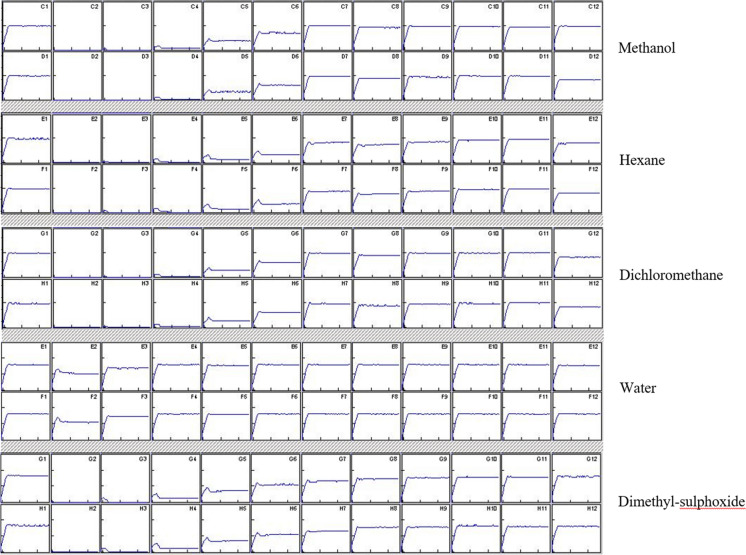
Kinetic diagram of the samples. Well1: control. Well2: highest, Well12: lowest concentrations. Light output is recorded in the first 30 s interval. In the control, light output remains even but in the actual samples light emittance is reduced, showing a concentration-dependent pattern. Each sample is analysed using two replicates (indicated by two rows in the diagram)

Figure 1 shows the light output diagrams of different extracts as continuously recorded during the first 30 secs of the measurement. Naturally, these results are difficult to quantify exactly but give a preliminary estimation of the toxicity of the samples. These diagrams are showing a similar pattern as the calculated EC_50_s: water extract is far less toxic than any of the other samples.

The chemical characterization of the extracts suggests that there are differences in the composition and amount of PAHs which can cause the variances in EC_50_ results. The PAH concentration without naphthalene is consistent with the results of toxicological measurements. The lowest PAH content and the highest EC_50_ were determined in the water extract, higher PAH concentration and lower EC_50_ were measured in methanol extract. The highest PAH amount and lowest EC_50_ values were measured in DCM, hexane and DMSO extracts.

Another important difference between extracts prepared with water and organic solvents is the profile of PAH isomers. In the aqueous extract, light molecular weight (LMW) PAHs are dominant, the only 4-ring PAH is fluoranthene with a low, 0.02 µg/mg concentration. On the other hand, diesel emissions contain higher molecular weight (HMW) 5- and 6-ring PAHs being responsible for carcinogenic effect (Islam et al., 2019; Kuo et al., 2012) (Table 2).

**Table 2:** Chemical characterization of extracts

Sample	Water	Methanol	DMSO	Hexane	DCM
Naphthalene (µg/mg)	7.340	5.430	7.478	5.004	5.728
2-metil—naphthalene (µg/mg)	0.190	0.142	0.291	0.130	0.147
1-metil—naphthalene (µg/mg)	0.090	0.063	0.166	0.058	0.066
Acenaphthylene (µg/mg)	0.000	0.008	0.012	0.006	0.004
Acenaphthene (µg/mg)	0.050	0.005	0.329	0.003	0.003
Fluorene (µg/mg)	0.010	0.008	0.013	0.011	0.008
Phenanthrene (µg/mg)	0.080	0.182	0.290	0.249	0.271
Anthracene (µg/mg)	0.014	0.016	0.034	0.026	0.033
Fluoranthene (µg/mg)	0.020	0.122	0.431	0.255	0.305
Pyrene (µg/mg)	0.000	0.165	0.523	0.366	0.450
Benz (a) anthracene (µg/mg)	0.000	0.017	0.120	0.075	0.082
Chrysene (µg/mg)	0.000	0.009	0.110	0.041	0.075
Benz (b) fluoranthene (µg/mg)	0.000	0.006	0.115	0.069	0.048
Benz (5) fluoranthene (µg/mg)	0.000	0.020	0.055	0.043	0.031
Benz (e) pyrene (µg/mg)	0.000	0.004	0.068	0.044	0.029
Benz (a) pyrene (µg/mg)	0.000	0.006	0.042	0.026	0.031
Indeno (1.2.3-cd) pyrene (µg/mg)	0.000	0.000	0.007	0.004	0.002
Dibenz (a. h) anthracene (µg/mg)	0.000	0.000	0.001	0.000	0.000
Benz (g. h. i) perylene (µg/mg)	0.000	0.000	0.001	0.001	0.001
Total naphthalene	7.620	5.635	7.935	5.192	5.941
Total PAHs	**0.174**	**0.568**	**2.151**	**1.219**	**1.372**
Total PAHs + naphthalene	7.794	6.203	10.086	6.411	7.313

Bold values are total PAH concentrations without naphthalene

The fact that aqueous extract would pose relatively lower toxicity is in line with other studies. Verma et al. (2012) measured ROS-generating potential of PM by the DTT assay. Response was significantly higher for the methanol extract. Płaza et al. (2005) compared the efficiency of bioremediation processes on selected biopiles. Microtox assays were carried out in DMSO/H_2_O and DCM/DMSO soil extracts, the latter showing considerably higher toxicity for all samples. Aqueous elutriates are generally supposed to underestimate the quantity and effect of bioavailable contaminants (Selivanovskaya et al., 2010).

Bioaccessibility is considered an important factor when potential impacts are to be evaluated (Thums et al., 2008; Turner, 2011) or regulatory frameworks for assessment protocols are addressed (Kim et al., 2015). Several studies have supported the strong influence of bioavailability of particulate-bound compounds on ecotoxic impact (Čvančarová et al., 2013; Jan et al., 2018; Sah et al., 2019; Varshney et al., 2016).

Comparing direct contact tests with elutriates, contact biotests are definitely suggested to determine the bioavailable fractions of substances and exposure pathways (Ivask et al., 2001). Tositti et al. (2018) applied the direct contact test version of the bioluminescent bacteria assay on PM10 samples and found significant correlation between chemical analysis and bioluminescence inhibition.

Unfortunately, there are very few studies available comparing the toxicity of different extracts and whole-medium using the same test organism. When risk assessment of solid media (sediment, soil, compost, etc.) is undertaken, the majority of the studies employ a whole battery of bioassays to represent the expected response of a simplified ecosystem (Juvonen et al., 2000). Lors et al. (2011) compared solid and liquid-phase bioassays using ecoscores derived from a wide range of bioassays for the assessment of contaminated soils and concluded that solid-phase tests had higher sensitivity. In a study of Tuikka et al. (2011), however, a wide range of direct contact tests was used to characterise sediment toxicity, including the kinetic *V. fischeri* assay. This assay proved sensitive for discriminating the contaminated sediments.

In some studies, however, different phases of the same sediment or soil sample were evaluated by the *V. fischeri* bioassay. Davoren et al. (2005) used the Microtox and Solid Phase Microtox tests to rank estuarine sediment samples. The solid phase test detected measurable toxicity for all samples, while liquid phase tests were much less sensitive: no toxicity was detected for elutriate samples and pore water samples were toxic only in one case. Similar results are given by Klimkowicz-Pawlas et al. (2019) when elutriates were tested by Microtox 81.9% Screening test and SPT-Microtox was used for testing solid samples. The solid phase Microtox® assay was more sensitive than saline extract Microtox assay in the study of Acheson et al. (2004) when soils contaminated with polycyclic aromatic hydrocarbons (PAHs) were investigated.

Gonzalez-Merchan et al. (2014) characterised the solid phase (whole sediment) and interstitial water phase of sediments from stormwater retention basins using the same Microtox protocols. In case of all samples, the solid phase test detected significantly higher ecotoxicity. Similar results were gained when ecotoxicity of a restored wetland was evaluated (Paulovits et al., 2012). Three different phases were tested using the kinetic luminescent bacteria test (ISO 21338:2010): whole sediment, pore water and elutriate. For every sampling site, direct tests showed considerably higher toxicity than elutriates, and no toxicity was detected in the pore water samples. Leitgib et al. (2007) applied bacterial assays (the *V. fischeri* bioluminescence inhibition and the *Azomonas agilis* dehydrogenase activity tests) for soil toxicity assessment, evaluating both direct contact and water extract toxicity. Direct contact tests showed higher impact for the majority of the samples.

Hursthouse and Kowalczyk (2009) discuss that when assessing the fate and effect of toxic pollutants, one of the major uncertainties lies in how to represent real-world conditions during experiments. Direct contact test seem better representatives considering the nature of exposure used.

Comparing organic solvents, DCM is generally applied to extract PAHs (Masood et al., 2018). It was also used in the study of Soriano et al. (2020) to assess genotoxic and mutagenic potential of soluble organic material extracted from PM emission of a diesel engine run on different alternative and/or renewable fuels and in the study of Morakinyo et al. (2020) to evaluate carcinogenic and mutagenic risks of PM2.5-bound PAHs. Jing et al. (2019) measured the residual toxicity of eight common extraction solvents in a study evaluating PM2.5 related biotoxicity in Shanghai. They found that luminescence intensity was unaffected when methanol, 1/1 N-hexane/dichloromethane, N-hexane and dichloromethane were used but the commonly used solvent, dimethyl-sulphoxide caused slight light inhibition. Based on the results of the study, considering both residual toxicity and the polarity of extraction solvents, N-hexane/dichloromethane, methanol, and ultrapure water were suggested for the extraction PM_2.5_. samples for the bioluminescent bacterium test.

As concluding remarks, we can say that while these studies refer to soil or sediment evaluation, our study is the first to compare the toxicity of different elutriates to a direct contact test. The benefit of the ‘whole-aerosol’ testing procedure is that test bacteria are in fact direct contact with particles. As such, the direct contact protocol intends to mimic environmentally realistic exposure route (exposed cells/organisms physically meet contaminated particles). Our results prove that its sensitivity equals the conventional ISO 11348 standard in cases when extracts are prepared with the application of ‘aggressive’ organic solvents such as methanol, DCM or hexane. The sample preparation protocol is repeatable, fulfilling basic quality assurance criteria, as such, interstudy comparisons can be carried out.

However, the ISO 11348 standard and sample preparation techniques based on this protocol have been much more widely used than the kinetic version of the *V. fischeri* bioluminescence inhibition assay. Altogether, based on extraction efficiency, luminescence inhibition and residual toxicity, DCM and methanol seem the most potent organic solvent for preparing PM samples. Considering the outcome of both analytical and ecotoxicological measurements, the direct contact test might be interchangeable with results gained for these extracts, taking into consideration the availability of laboratory equipment and/or protocols.

## Data Availability

Data generated during the study are included in the manuscript.
